# Alveolar Bone Density Reduction in Rats Caused by Unilateral Nasal Obstruction

**DOI:** 10.4274/balkanmedj.galenos.2019.2018.12.11

**Published:** 2019-11-28

**Authors:** Xue Wang, Yongge Cao, Zhenhua Liu, Zihan Wang, Xiaoying Chu, Lei Wang, Xuanxuan Hu, Han Zhao, Zhanqiu Diao, Fengting Peng, Hui Ye, Zhensheng Cao

**Affiliations:** 1 Department of Biochemistry, School of Basic Medical Sciences, Wenzhou Medical University, Wenzhou, Zhejiang, China; 2 Haiyuan College, Kunming Medical University, Kunming, Yunnan, China; 3 School of Stomatology, Wenzhou Medical University, Wenzhou, Zhejiang, China; 4 School of the Second Clinical Medical Sciences, Wenzhou Medical University, Wenzhou, Zhejiang, China

**Keywords:** Bone mineral density, nasal obstruction, rats, receptor activator of nuclear factor-κB, receptor activator of nuclear factor-κB ligand

## Abstract

**Background:**

Oral breathing can cause morphological changes in the oral and maxillofacial regions.

**Aims:**

To investigate whether oral breathing affected structural changes in bone tissues.

**Study Design:**

Animal experimentation.

**Methods:**

A total of 48 8-day-old male Sprague−Dawley rats were divided into two groups: a breathing group and a sham (control) group. All Sprague−Dawley rats were killed at 7 weeks after unilateral nostril obstruction modeling. Then, structural changes in bone tissues were detected by micro-computed tomography, and the expression levels of receptor activator of nuclear factor-κB, osteoprotegerin, and receptor activator of nuclear factor-κB ligand in the signal pathway of bone metabolism within the local alveolar bone and serum of rats were detected by reverse transcription-quantitative polymerase chain reaction and Western blotting.

**Results:**

The results showed that receptor activator of nuclear factor-κB ligand and receptor activator of nuclear factor-κB levels in bone tissues and serum in the oral breathing group were higher than those in the control group [Maxillary alveolar bone: receptor activator of nuclear factor-κB ligand (pRNA=0.009, pprotein=0.008), receptor activator of nuclear factor-κB (pRNA=0.008, pprotein=0.009); Mandibular alveolar bone: receptor activator of nuclear factor-κB ligand (pRNA=0.047, pprotein=0.042), receptor activator of nuclear factor-κB (pRNA=0.041, pprotein=0.007); Serum: receptor activator of nuclear factor-κB ligand (pRNA<0.001, pprotein<0.001), receptor activator of nuclear factor-κB (pRNA<0.001, pprotein<0.001)], along with decreased osteoprotegerin expression (Maxillary alveolar bone: pRNA=0.038, pprotein=0.048; Mandibular alveolar bone: pRNA<0.001, pprotein<0.001; Serum: pRNA=0.009, pprotein=0.006) and elevated receptor activator of nuclear factor-κB ligand/osteoprotegerin. Micro-computed tomography analysis indicated a significant difference in the level of bone volume fraction, as well as trabecular thickness in maxillary alveolar bone between the experimental and control groups (p=0.049, p=0.047). Meanwhile, trabecular thickness, and cortical thickness levels in mandibular alveolar bone also differed significantly between the experimental and control groups (p=0.043, p=0.024).

**Conclusion:**

Structural changes of the respiratory system affect the alveolar bone structure and unilateral nasal obstruction may lead to a change in regional specific bone density.

Oral breathing has an important impact on the growth and development of craniofacial structures (1,2,3,4,5). Animal experiments demonstrated that changes in breathing patterns from the nasal cavity to the oral cavity can induce changes in craniofacial muscle activity (6,7,8,9,10,11). Recent studies have focused on the effect of oral breathing on the morphology of maxillofacial (12,13). However, the adaptive structural component of alveolar bone remains unclear.

In orthodontic patients, many factors contribute to the success of orthodontic microimplants, among which cortical bone thickness and bone density are important factors affecting the success of orthodontic microimplants (14,15,16,17,18,19,20,21,22,23,24).

Oral breathing is speculated to be related to a decrease in bone mineral density in local alveolar bone. Nonetheless, further studies are needed to determine whether the poor bone quality of local alveolar bone can be attributed to oral breathing.

In this study, we hypothesized that after unilateral nostril obstruction, changes in resistance in the nasal cavity to the oral passage may change the cancellous bone of the alveolar bone and the bone mass of the cortical bone due to local and systemic changes, thereby reducing the density of the alveolar bone.

## MATERIALS AND METHODS

### Animals and feeding

Lactating Sprague−Dawley (SD) female rats (250-300 g) and their 8-day-old neonatal offspring (18-20 g) were purchased from the Shanghai Laboratory Animal Center in the Chinese Academy of Sciences (Shanghai, China). All rats were fed with standard rodent food and given free access to water in an specific-pathogen-free animal room with constant temperature and a 12-h light–dark cycle. All animal experiments were reviewed and approved by the animal and ethical review committee of committee of Wenzhou Medical University (Wenzhou Medical University Policy and Welfare Committee; File ID No: WMU-2011-AP-0013).

### Reagents

TRIzol reagent, DNaseI enzyme, Moloney Murine Leukemia Virus reverse transcriptase, and SYBR Green positron emission tomography Master Mix were purchased from Invitrogen (Carlsbad, CA, USA). The BCA protein analysis reagent kit was provided by Pierce (Rockford, USA). Polyvinylidene fluoride was provided by Bio-Rad Laboratories (Hercules, USA). Rabbit anti-human osteoprotegerin (OPG) and receptor activator of nuclear factor-κB ligand (RANKL) were purchased from Novus Biologicals (Littleton,USA), rabbit anti-human receptor activator of nuclear factor-κB (RANK), β-Actin as well as goat anti-rabbit IgG horseradish peroxidase were provided by Cell Signaling Technology (Boston, USA). The ECL reagent kit was purchased from Amersham Pharmacia Biotech (Piscataway, USA), and all other reagents were of analytical grade.

### Establishment of model rats and sample collection

**Figure 1:**
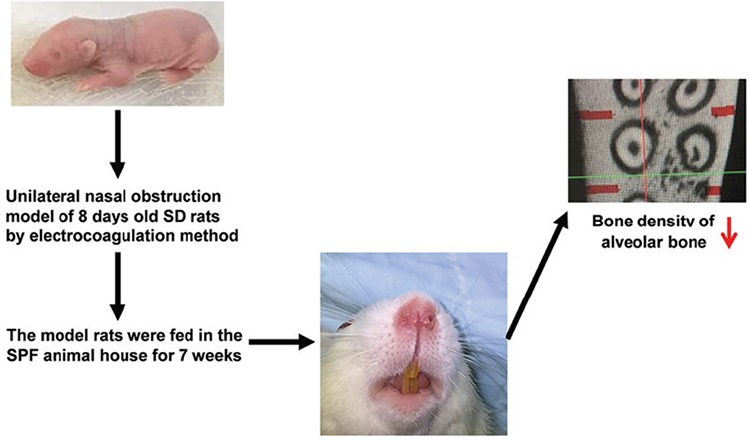
Establishment of unilateral nasal obstruction in SD model rats: Rats were raised in an SPF animal house (breastfeeding in the early stage; standard rodent food and free access to water in the later stage) and then sacrificed at 7 weeks after modeling. The alveolar bone of each rat was quickly collected and immersed in an EP tube containing 4% paraformaldehyde for subsequent bone mineral density analysis. SD: Sprague−Dawley; SPF: specific-pathogen-free

A total of 48 8-day-old male SD neonatal rats were randomly divided into three pairs: group 1 (maxillary alveolar bone of unilateral nostril electrocautery group) vs group 2 [maxillary alveolar bone of control group (sham group)]; group 3 (mandible alveolar bone of unilateral nostril electrocautery group) vs group 4 (mandible alveolar bone of control group); group 5 (serum of unilateral nostril electrocautery group) vs group 6 (serum of control group). There were 24 rats in each of the oral breathing group and sham group with four rats in each cage, which contained one adult breastfeeding SD female rat. In the oral breathing group (unilateral nostril electrocautery group, cages 1-6), the left nostrils of neonatal rats were blocked with a high-frequency electric knife (Figure 1). In the sham group (cages 7-12), the rats received no treatment. Both groups were breastfed in the early stage and were provided with standard rodent food and free access to water. All rats were sacrificed after 7 weeks of modeling. The maxillary and mandible alveolar bones were collected immediately and divided into three parts: the first part was soaked in TRIzol for subsequent reverse transcription-quantitative polymerase chain reaction analysis; the second part was preserved in liquid nitrogen for subsequent western blot analysis; and the third part was soaked in an EP tube with 4% paraformaldehyde for bone density analysis. Meanwhile, venous blood was collected from the neck of each rat and then centrifuged for 10 min at 4 °C. Afterwards, the serum was obtained by removing the supernatant and was preserved in liquid nitrogen.

### Measurement of bone density of SD rats

One-third of the collected maxillary and mandible alveolar bones were immediately soaked in an EP tube with 4% paraformaldehyde for subsequent analysis of bone density ***(See; Animals and feeding)***. Then, the maxillary and mandible alveolar bones were scanned by micro-computed tomography (PerkinElmer, Model Number: QuantumGX). Micro-computed tomography scanning and 3D reconstruction technology were used to scan the maxillary and mandible bones of rats and standard phantom simultaneously for computed tomography value correction. Quantitative analysis was achieved with system-provided Analyze 12.0 software. The scanning pixel size was 18 µm; the scanning voltage was 90 kV; the scanning current was 80 µA; the scanning mode was 360° rotation; the scanning time was 14 min; the scanning field of vision was 9 mm×9 mm×9 mm. Quantitative analysis of bone employed the following main measurement parameters: trabecular bone volume fraction (BV/TV), trabecular thickness (Tb.Th.), and cortical thickness (Cort.Th.) of maxillary and mandibular alveolar bone. Bone thickness and density were mainly tested in the alveolar bone cortex.

### Reverse transcription-quantitative polymerase chain reaction detection of genes related to bone mineral density

**Primer design and synthesis:** Sequences of OPG, RANK, and RANKL were obtained from the NCBI database (http://www.ncbi.nlm.nih.gov), and then Primer premier 6.0 software was used to design the upstream and downstream primers of the three genes by positron emission tomography. All primers were synthesized by the Beijing Genomics Institute (primer sequences are shown in Table 1).

**Total RNA extraction and quality detection:** TRIzol reagent was used to extract total RNA from the serum of SD rats in the experimental and control groups. Similarly, total RNA was extracted from the maxillary and mandible alveolar bones of SD rats. Subsequently, the quality of total extracted RNA was detected by 1% agarose gel electrophoresis and spectrophotometry.

**Reverse transcription-quantitative polymerase chain reaction, gel extraction, and cloning sequencing:** cDNA synthesis was completed with 2 μg of total RNA, and the 25 μL reverse transcriptional system was composed of the following components: 2 μL of real-time primers (10 μmol/L), 5 μL 5× Moloney Murine Leukemia Virus buffer, 1.25 mL dNTPs (10 nmol/L), 1 μL of reverse transcriptase (200 U/μL), and 0.6 μL of RNA enzyme inhibitor (40 U/μL); and RNase-free water to a total volume of 25 μL. The reaction conditions were set at 16 °C for 20 min, 42 °C for 30 min, and 85 °C for 10 min to inactivate the reverse transcriptase, which was then preserved at 4 °C.

**The quantitative polymerase chain reaction amplification system (50 μL) was made up of the following components:** 5 μL of cDNA template, 0.2 μL of upstream and downstream primers (20 μM), 25 μL of 2× SYBR Green positron emission tomography Master Mix, and ddH_2_O. Positron emission tomography amplification was carried out according to the following setting parameters: pre-denaturation at 95 °C for 3 min, followed by 40 cycles (denaturation at 95 °C for 12 s, annealing at 62 °C for 40 s, prolongation at 72 °C for 45 s), and then at 72 °C for 10 min. The mRNA levels of OPG, RANK, and RANKL were quantified relatively, and Glyceraldehyde-3-phosphate dehydrogenase was used as an internal reference.

Electrophoreses of quantitative positron emission tomography products was conducted in 4% agarose, and gel extraction was performed according to the instructions of the general Tiangen agarose DNA recovery kit. Extracted fragments were connected with PMD-19T carrier in accordance with the manufacturer’s instructions. Then, DH5α competent *Escherichia coli* cells were transformed with the fragments. Furthermore, eight bacterial colonies were selected for liquid culture by using LA agar plate culture medium. Positive clones identified by positron emission tomography were delivered to the Beijing Genomics Institute for sequencing.

### Western blot analysis

Total protein in maxillary and mandible alveolar bones or serum of rats was extracted from the experimental and control groups. The protein concentration was determined by means of the BCA protein analysis kit (Pierce, Chemical Co., USA). Furthermore, 2 µL of 5× SDS-PAGE loading buffer was added to the 8 µL sample, with heat treatment at 100 °C for 5 min and centrifugation at 10,000 rpm for 1 min to remove the insoluble precipitate. The sample was separated with 8% and 10% SDS-PAGE. The amount of sample per well was 10 µL. After electrophoresis, polyvinylidene fluoride membrane was soaked in methanol for 10 s. The gel and polyvinylidene fluoride membrane were soaked in rapid electrophoretic buffer for 10 min, and then the sandwich transfer structure was prepared, followed by transfer with the wet phase-inversion method (transferring conditions: 200 mA for 25 min). After the transfer was complete, the polyvinylidene fluoride membrane was soaked in methanol for 10 s and then incubated at 4 °C overnight with the primary antibody at an appropriate dilution. The sample was cleaned 5 times with 1× TBST (10 min each time). Transmembrane protein was incubated with enhanced chemiluminescent buffer and then developed with X-ray film. The density of immunoreactive bands was analyzed by using version 1.61 NIH image analysis software (National Institutes of Health, Bethesda, USA). OPG (or RANK or RANKL) protein was quantified with β-actin as the internal reference.

### Statistical analysis

SPSS 22.0 software package was used for statistical analysis. The data were expressed as mean ± standard deviation. T test was used for comparison between the two groups (the details are as follows: group 1 vs group 2, group 3 vs group 4, group 5 vs group 6). P<0.05 meant that the difference was statistically significant.

## RESULTS

### Bone density analysis

**Figure 2:**
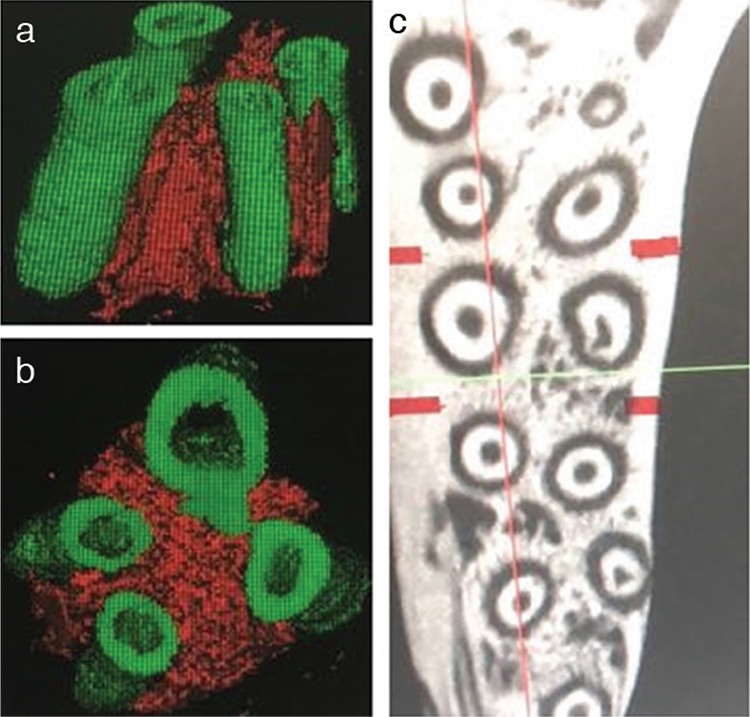
**a-c.** Bone density analysis of maxillary and mandibular alveolar bones: Sagittal view (a), horizontal view (b), and axial cross-section images of selected regions of interest in the alveolar bone adjacent to the apical third of the mesial root of the maxillary left first molar (c). Red and green represent trabecular bone and tooth root, respectively, in (a) and (b); red represents cortical thickness in (c).

Figure 2a, b, and c display the sagittal view, horizontal view, and cortical thickness, respectively. Our study detected the BV/TV, Tb.Th., and Cort.Th. of maxillary and mandibular alveolar bone. As shown in Table 2, a significant difference was found in the level of BV/TV, as well as Tb.Th., between maxillary alveolar bone of the experimental and control groups (p=0.049, p=0.047, respectively). Meanwhile, Tb.Th. and Cort.Th. levels in mandibular alveolar bone of the experimental group were also significantly different from those in the control group (p=0.043, p=0.024). However, no significant difference was found in the level of Cort.Th in maxillary alveolar bone between the experimental and control groups (p=0.072). No significant difference was found in the level of BV/TV in mandibular alveolar bone between the experimental and control groups (p=0.076). Thus, oral breathing caused by nasal obstruction did not lead to a decrease in alveolar bone density and bone strength in SD mice.

### RNA expression levels of bone-density-related genes

**Figure 3:**
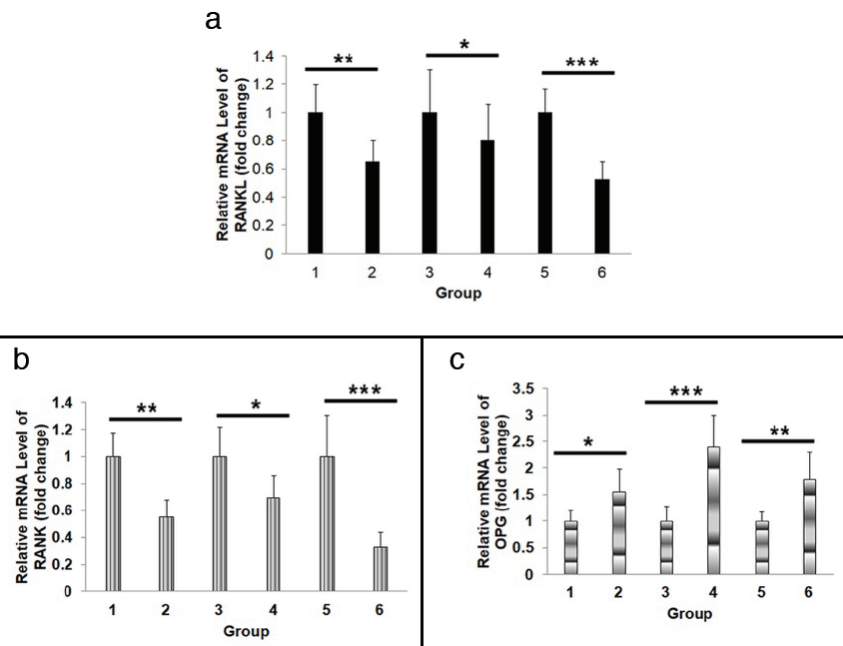
**a-c.** RNA expression level of bone-density-related genes. RT-qPCR was used to detect the expression of RANKL (*p=0.047, **p=0.009, and ***p<0.001), RANK (*p=0.041, **p=0.008, and ***p<0.001), and OPG (*p=0.038, **p=0.009, and ***p<0.001) in maxillary and mandible alveolar bones as well as in the serum (a-c). The values are expressed as mean ± standard deviation from three independent experiments. Note: 1 (maxillary alveolar bone of unilateral nostril electrocautery group) vs 2 (maxillary alveolar bone of the control group), 3 (mandible alveolar bone of unilateral nostril electrocautery group) vs 4 (mandible alveolar bone of the control group), and 5 (serum of unilateral nostril electrocautery group) vs 6 (serum of the control group). OPG: osteoprotegerin; RANK: receptor activator of nuclear factor-κB; RANKL: receptor activator of nuclear factor-κB ligand; RT-qPCR: reverse transcription-quantitative polymerase chain reaction

The sequencing results provided by the Beijing Genomics Institute were considered completely correct. Reverse transcription-quantitative polymerase chain reaction results showed that the expression levels of RANKL and RANK in maxillary alveolar bone, mandible alveolar bone, and serum were higher in the unilateral nostril electrocautery group than in the control group: 1 (maxillary alveolar bone of unilateral nostril electrocautery group) vs 2 (maxillary alveolar bone of the control group), RANKL (**p=0.009), RANK (**p=0.008); 3 (mandible alveolar bone of unilateral nostril electrocautery group) vs 4 (mandible alveolar bone of the control group), RANKL (*p=0.047), RANK (*p=0.041); and 5 (serum of unilateral nostril electrocautery group) vs 6 (serum of the control group), ***p<0.001) (Figure 3a, b). On the contrary, the expression levels of OPG in the maxillary alveolar bone, mandible alveolar bone, and serum were lower in the experimental group than that in the control group: 1 (maxillary alveolar bone of unilateral nostril electrocautery group) vs 2 (maxillary alveolar bone of the control group), *p=0.038; 3 (mandible alveolar bone of unilateral nostril electrocautery group) vs 4 (mandible alveolar bone of the control group), ***p<0.001; 5 (serum of unilateral nostril electrocautery group) vs 6 (serum of the control group), **p=0.009) (Figure 3c).

### Protein expression level of bone-density-related genes

**Figure 4:**
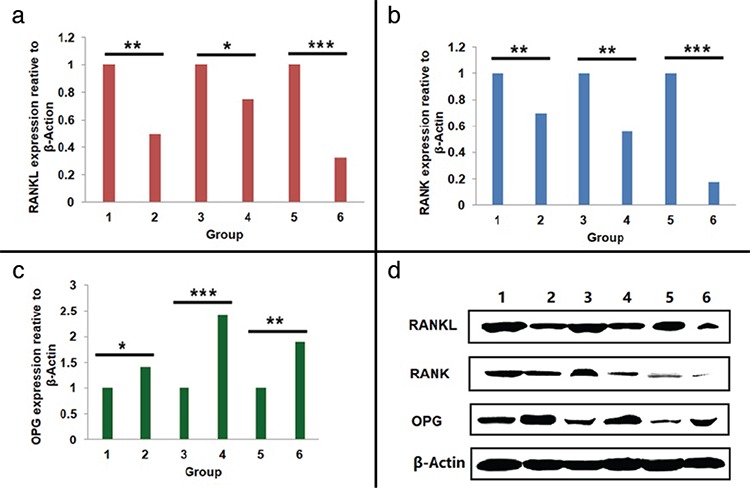
**a-d.**Protein expression level of bone-density-related genes. Western blotting was used to detect the expression of RANKL (*p=0.042, **p=0.008, and ***p<0.001), RANK (**p_12_=0.009, **p_34_=0.007, and ***p<0.001), and OPG (*p=0.048, **p=0.006, and ***p<0.001) in maxillary and mandible alveolar bones as well as in the serum. The values are expressed as mean ± standard deviation from three independent experiments (a-d). Note: 1 (maxillary alveolar bone of unilateral nostril electrocautery group) vs 2 (maxillary alveolar bone of the control group), 3 (mandible alveolar bone of unilateral nostril electrocautery group) vs 4 (mandible alveolar bone of the control group), and 5 (serum of unilateral nostril electrocautery group) vs 6 (serum of the control group). OPG: osteoprotegerin; RANK: receptor activator of nuclear factor-κB; RANKL: receptor activator of nuclear factor-κB ligand

Western blot results indicated that RANKL and RANK levels in maxillary alveolar bone, mandible alveolar bone, and serum were higher in the experimental group than in the control group: 1 (maxillary alveolar bone of unilateral nostril electrocautery group) vs 2 (maxillary alveolar bone of the control group), RANKL (**p=0.008), RANK (**p=0.009); 3 (mandible alveolar bone of unilateral nostril electrocautery group) vs 4 (mandible alveolar bone of the control group), RANKL (*p=0.042), RANK (**p=0.007); and 5 (serum of unilateral nostril electrocautery group) vs 6 (serum of the control group), ***p<0.001 (Figure 4a, b, d). In contrast, the expression levels of OPG in the maxillary alveolar bone, mandible alveolar bone, and serum were lower in the experimental group than that in the control group: 1 (maxillary alveolar bone of unilateral nostril electrocautery group) vs 2 (maxillary alveolar bone of the control group), *p=0.048; 3 (mandible alveolar bone of unilateral nostril electrocautery group) vs 4 (mandible alveolar bone of the control group), ***p<0.001; and 5 (serum of unilateral nostril electrocautery group) vs 6 (serum of the control group), **p=0.006 (Figure 4c, d).

### Possible mechanism of alveolar bone osteoporosis

**Figure 5:**
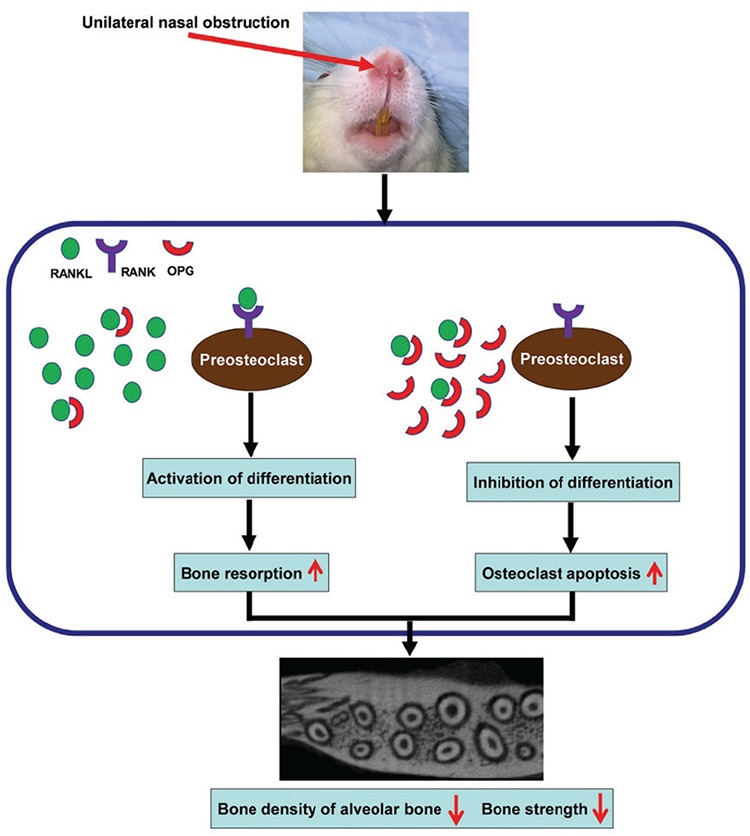
Possible mechanism of alveolar bone osteoporosis induced by nasal obstruction in SD rats. OPG: osteoprotegerin; RANK: receptor activator of nuclear factor-κB; RANKL: receptor activator of nuclear factor-κB ligand; SD: Sprague−Dawley

As shown in Figure 5, when the amount of RANKL produced in the microenvironment around the osteoclastic precursor is significantly higher than that of OPG (natural antagonist of RANKL), RANKL combines with the RANK expressed in the osteoclastic precursor, thereby disturbing the balance of bone remodeling, which is beneficial to the formation of osteoclasts and the activation of bone absorption. In contrast, when the ratio of RANKL/OPG is reduced, OPG binds to RANKL, which prevents the binding of RANKL and RANK, thus inhibiting osteoclast formation. Meanwhile, inhibition of OPG binding to RANKL and RANK also promotes apoptosis in activated mature osteoclasts. Therefore, the relative ratio of RANKL/OPG determines the speed and intensity of osteoclast-mediated bone resorption.

## DISCUSSION

Oral breathing may affect facial and occlusal development in early childhood. Oral breathing children are more likely to present reduced posterior facial height and a narrow maxilla (23). Further studies are necessary to explore the changes caused by oral breathing.

It is difficult to recruit clinical patients as oral breathing models. In this study, an oral breathing model of SD neonatal rats was constructed by means of electrocoagulation for unilateral nostril obstruction, with which bone density and RANK, RANKL, and OPG levels can be detected easily.

In this study, the OPG/RANKL ratio was suggested to regulate normal bone metabolism. Accordingly, the relative ratio of RANKL/OPG determines the speed and intensity of bone resorption.

Three proteins (RANK, RANKL, and OPG) and their RNA expression levels were examined. Reverse transcription-quantitative polymerase chain reaction results revealed that the expression levels of RANKL and RANK in maxillary alveolar bone, mandible alveolar bone, and serum were all higher in the experimental group than those in the control group, whereas the opposite was found regarding the expression level of OPG. These results were consistent with the results of western blot analysis. Thus, the increase in RANK and RANKL expression increases the activity of osteoclasts, but the decrease in OPG expression inhibits the full realization of the effect of osteoclasts, resulting in excessive bone absorption and probably a decrease in bone mineral density in alveolar bone.

As shown in Table 2, micro-computed tomography analysis indicated that a significant difference was found in the level of BV/TV, as well as Tb.Th., between maxillary alveolar bone of the experimental and control groups (p=0.049, p=0.047). Meanwhile, Tb.Th. and Cort.Th. levels in mandibular alveolar bone of the experimental group were significantly different from those in the control group (p=0.043, p=0.024). However, no significant difference was found between level of Cort.Th in maxillary alveolar bones (p=0.072). In addition, no significant difference was found between levels of BV/TV in mandibular alveolar bones (p=0.076). During our previous modeling by electrocoagulation, we found that 8-day-old SD rats with bilateral nasal obstruction had a much higher mortality rate than those with unilateral nasal obstruction. Therefore, 8-day-old SD rats with unilateral nasal obstruction were used in this study.

Our results indicate that oral breathing leads to a decrease in the bone density of compact and cancellous bone in the maxillary and mandible alveolar bones of SD rats. However, more intensive research on the mechanism is required. Previous research demonstrated that oral breathing induces chewing activity (24), but whether the decrease in chewing activity will reduce the bone density of alveolar bones remains unclear. Oral breathing is known to affect craniofacial muscle activity (6,8,9,10). Nonetheless, further studies are needed to explore the relationship between craniofacial muscle activity and the density of alveolar bones.

Prior experiments have shown that intermittent hypoxia can cause a reduction in bone mass, and the mechanism of the OPG/RANKL/RANK system will contribute to the exploration of the mechanism of bone mass decline in alveolar bone (12).

Previous studies documented that breathing can affect occlusion (25). The impact of breathing on the maxillofacial muscles has also been demonstrated, but the impact of breathing patterns on the jaw is not clear (6,7). RANK, RANKL, and OPG protein levels determined by western blot analysis and their corresponding RNA levels determined by reverse transcription-quantitative polymerase chain reaction also provided insight into the possible mechanism of decline in bone mineral density. The change in airflow from nasal passage to oral passage after unilateral nasal obstruction might lead to a change in the structure of cancellous bone and the cancellous bone in alveolar bones. In addition, alveolar bone remodeling in patients involving oral breathing, such as the stability of implant anchorage, may require consideration of changes in the strength of alveolar bone. Our current research could provide guidance for the selection of implant anchorage in clinical practice. For oral breathing patients, locations with higher bone density are better for implanting anchorage.

The current experimental results cannot be directly applied to humans. However, our experiment has shown that changes in airway structure may affect alveolar bone structure, which may be related to local and systemic factors.

This study confirmed that structural changes of the respiratory tract affect alveolar bone structure, and unilateral nostril obstruction may lead to a change in regional specific bone density during growth and development of the maxillary and mandible. In addition, changes in alveolar bone structure may be related to local and systemic factors.

## Figures and Tables

**Table 1:**
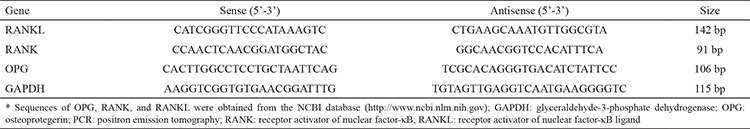
Sequences of primers used in PCR assay

**Table 2:**
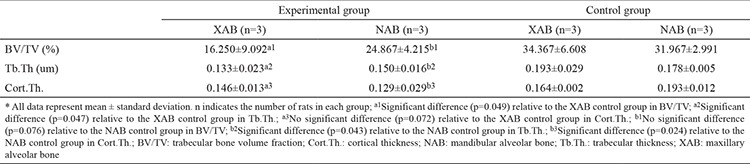
Effect of unilateral nasal obstruction on the microstructural data obtained through microcomputed tomography
